# PD218 Quantitative Analysis Of The Policy Text For Free Training Of Rural Order-Oriented Medical Students In China

**DOI:** 10.1017/S0266462324004380

**Published:** 2025-01-07

**Authors:** Xiaona Li, Min Gao, Jiaxian Shao, Yuncong Yu, Wenqiang Yin, Zhongming Chen

## Abstract

**Introduction:**

To speed up the construction of grassroots medical and health teams in China, free training of rural order-oriented medical students was launched in June 2010. Based on the theory of policy tools, a quantitative analysis of policy texts at the national level was conducted to explore the use of policy tools and to put forward corresponding suggestions for adjustments.

**Methods:**

From January to February 2023, the research team searched the Peking University Treasure Database and the official websites of the State Council, the National Health Commission, the Ministry of Education, and other ministries for national policy documents related to free training of order-oriented medical students published from June 2010 to May 2023. A policy tool and policy target analysis framework were used to quantitatively analyze the policy documents.

**Results:**

A total of 16 policy documents were included and 213 policy provisions were extracted. From the perspective of policy tools, the proportion of policy provisions using imperative policy tools was the highest (63.4%), followed by advisory policy tools (18.8%). and reward-based policy tools (13.6%). Functional expansion tools (2.8%) and authoritative restructuring tools (1.4%) accounted for a relatively low proportion. The institutional education stage is the main policy target, with provisions accounting for 75 percent (162 articles), followed by the continuing education stage (17.6%; 38 articles), and the postgraduate education stage (7.4%; 16 articles).

**Conclusions:**

The distribution of policy tools for the free training policy of rural order-oriented medical students in China needs to be balanced, and the internal combination of the same policy tools needs to be optimized. The policy targets were mainly concentrated in the education stage of universities.

